# Retinal Pigment Epithelial Tears after Ex-PRESS Filtration Surgery in a Glaucoma Patient with a History of Ischemic Optic Neuropathy

**DOI:** 10.1155/2023/6645156

**Published:** 2023-10-25

**Authors:** Mamiko Takemoto, Yuta Kitamura, Masato Kakisu, Daisuke Shimizu, Takayuki Baba

**Affiliations:** ^1^Department of Ophthalmology and Visual Science, Chiba University Graduate School of Medicine, Japan; ^2^Department of Ophthalmology, International University of Health and Welfare Narita Hospital, Japan; ^3^Department of Ophthalmology, Japanese Red Cross Narita Hospital, Japan

## Abstract

**Background:**

To describe a case of retinal pigment epithelial tears (RPE tears) and serous retinal detachment (SRD) after Ex-PRESS filtration surgery for primary open-angle glaucoma (POAG) combined with ischemic optic neuropathy. *Case Presentation*. This case report involved a 69-year-old woman who underwent Ex-PRESS filtration surgery for right POAG. She had a history of systemic arteriosclerotic disease and subacute progressive visual field loss due to suspected ischemic optic neuropathy in her right eye. The right preoperative visual acuity was 0.7, and intraocular pressure (IOP) was 19 mmHg with maximum glaucoma eye drops. RPE detachment was not observed in the fundus. On day 9 after surgery, the IOP was 6 mmHg, and mild choroidal detachment was observed. On day 13, although IOP remained almost unchanged at 7 mmHg, bullous SRD was observed in the inferior retina, including the macula, and RPE tears were observed along the superior arcade vessel. While subretinal fluid gradually decreased with increasing IOP, tractional retinal folds persisted along the superior arcade, accompanied by macular degeneration.

**Conclusion:**

We experienced a case of RPE tears after Ex-PRESS filtration surgery. In addition to choroidal detachment in the setting of hypotony, a pathologic condition causing structural fragility of the RPE layer may contribute to the development of RPE tears.

## 1. Introduction

Retinal pigment epithelial tears (RPE tears) are commonly associated with exudative age-related macular degeneration, as well as with certain treatments such as antivascular endothelial growth factor (VEGF) drug therapy and photodynamic therapy [1, 2]. Other reported causes of RPE tears include central serous chorioretinopathy (CSC), retinal pigment striae, uveitis, myopia, choroidal tumors, trauma, and buckling surgery [3–9]. While choroidal detachment and hypotony maculopathy are well-known complications that occur in the fundus after glaucoma filtration surgery, RPE tears are extremely rare. Recently, there have been a few reports of RPE tears after filtration surgery; however, detailed pathogenesis and treatment have not yet been established [5, 10]. Since severe visual dysfunction may occur depending on the location and size of RPE tears, the identification of risk factors for this complication and the establishment of preventive methods and effective treatment strategies are needed. In this study, we report a case of RPE tears and serous retinal detachment (SRD) following Ex-PRESS filtration surgery in a patient with primary open-angle glaucoma (POAG) with a history of ischemic optic neuropathy.

## 2. Case Presentation

A 69-year-old woman who had been regularly visiting an ophthalmologist for diabetic retinopathy was diagnosed with POAG in both eyes 5 years ago. She was treated with glaucoma eye drops (a combination of dorzolamide hydrochloride and timolol maleate). During the same period, cataract surgery was performed in the right eye. Although intraocular pressure (IOP) remained around 15 mmHg in both eyes, the right central visual field defect gradually worsened over time. In six months prior to her referral to our hospital, there was a subacute deterioration of the visual field in the right eye. The patient noticed a sudden decline in vision in her right eye, without any eye pain. The patient was referred to our hospital for further examination. The patient had several systemic diseases including well-controlled diabetes mellitus, hypertension, hyperlipidemia, and arteriosclerosis obliterans. She had a history of a transient ischemic attack 10 years earlier, for which she was currently on antiplatelet medication. She was also diagnosed with chronic inflammatory demyelinating polyneuritis 15 years prior and had been treated with oral steroids for 3 years.

**Figure 1:**
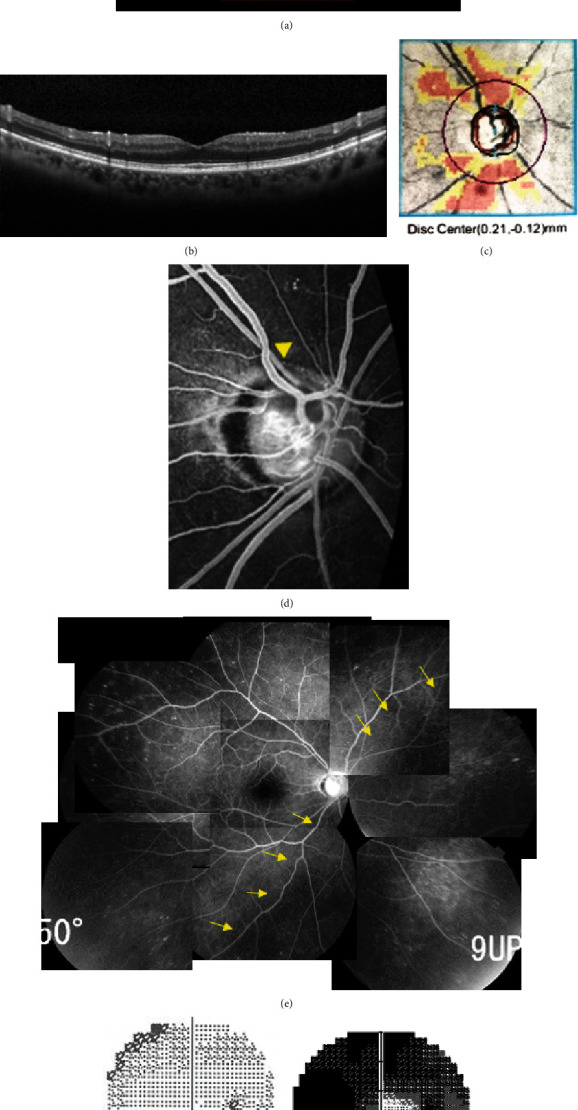
Findings at the initial presentation of the patient. Color fundus photograph showed slightly paled optic disc and large cupping of optic disc (a). Vertical scan of optical coherence tomography (OCT) showed normal findings without any pigment epithelial detachment (PED) and choroidal neovascularization (b). OCT imaging of the circumpapillary retinal nerve fiber layer (cpRNFL) deviation map in the right eye showing thinning of the cpRNFL on both the superior and inferior sides (c). Fluorescein angiography showed partial delayed filling in the optic disc at superior and nasal side (arrowhead) (d) and delayed filling of the choroidal perfusion around the nasal-inferior region, including near the optic disc (arrow) (e). A Humphrey visual field test performed at our hospital one month after the initial visit revealed a severe visual field defect (g) that was much worse than the result observed at the previous hospital nine months earlier (f).

At the time of her presentation, best-corrected visual acuity (BCVA) was 0.7 in the right eye and 0.9 in the left eye. The IOP was 25 mmHg in the right eye and 18 mmHg in the left eye. The central corneal thickness was 593 *μ*m in the right eye and 568 *μ*m in the left eye, and the axial length was 22.05 mm in both eyes. Both eyes were pseudophakic. Fundus examination revealed that both eyes had large optic disc cuppings predominantly in the right eye and significant thinning of the rim in the right eye. No signs of papilledema were observed (Figure 1(a)). Optical coherence tomography (OCT) showed no retinal pigment epithelial detachment (PED) or choroidal neovascularization in the macula (Figure 1(b)), but there was scattered drusen in the peripheral retina. OCT imaging of the circumpapillary retinal nerve fiber layer (cpRNFL) deviation map demonstrated thinning of the cpRNFL on both the superior and inferior sides (Figure 1(c)). Fluorescein angiography (FAG) showed partial delayed filling in the optic disc at superior and nasal sides and delayed filling of the choroidal perfusion around the nasal-inferior region, including near the optic disc (Figures 1(d) and 1(e)). Visual field testing was performed using a Humphrey visual field analyzer which showed a significant worsening during recent 9 months (Figures 1(f) and 1(g)).

Since the patient had a positive right relative afferent pupillary defect and a mild decrease in central flicker value in the right eye (26.3 Hz in the right eye and 32.8 Hz in the left eye), a contrast-enhanced magnetic resonance imaging was performed to examine the development of optic neuritis. However, no active optic neuritis was detected. Based on the history of sudden painless visual decline, subacute progression of visual field defects, presence of systemic arteriosclerotic diseases (hypertension, hyperlipidemia, diabetes mellitus, and arteriosclerosis obliterans), and a history of transient ischemic attacks, along with findings of delayed perfusion of optic nerve disc and choroid on FAG, ischemic optic neuropathy was suspected to have developed during glaucoma treatment in the right eye. Although both IOPs remained at approximately 15 mmHg with the maximum tolerated medical therapy, the residual visual field in the right eye further deteriorated. Owing to the worsening of the central visual field defects, we determined that further IOP reduction was necessary, and Ex-PRESS filtration surgery was performed in the right eye.

**Figure 2:**
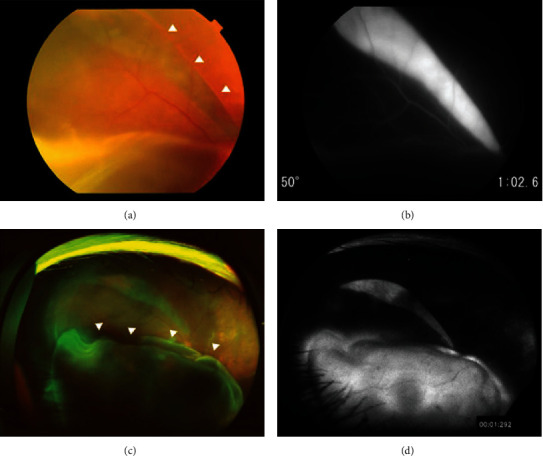
Findings at the onset of RPE tears on postoperative day 13. Color fundus photograph revealed RPE tears along the superior arcade vessel (arrowhead) (a). Fluorescein angiography (FAG) showed window defects corresponding to the site of RPE tears (b). Wide-field fundus photograph showed bullous macula off retinal detachment inferiorly (arrowhead) (c). Wide-field FAG revealed window defects corresponding to the site of RPE tears extending from near the optic nerve disc to the superior-temporal peripheral area (d).

**Figure 3:**
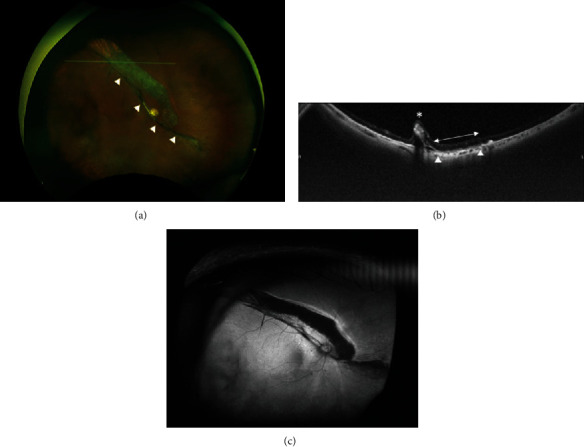
Wide-field fundus photograph and optical coherence tomography (OCT) imaging 12 months after Ex-PRESS filtration surgery. Fundus photograph showed that although retinal detachment almost recovered, widened RPE tears and subretinal proliferative tissue remained superior to the posterior pole (arrowhead) (a). OCT imaging corresponding to the line in the fundus photograph revealed an RPE defect (arrow) and partial traction of the retina due to subretinal proliferative tissue (asterisk) (b). Fundus autofluorescence (FAF) showed hypofluorescence corresponding to the area of RPE tears (c).

The surgery was completed without any complications. Laser suture lysis was performed, and the patient was discharged from the hospital on the fourth postoperative day with an IOP of 3 mmHg in the right eye. On the ninth postoperative day, choroidal detachment was observed in the superior peripheral retina at an IOP of 6 mmHg. The anterior chamber was slightly shallow, and atropine sulfate hydrate eye drops were initiated in the right eye. On the thirteenth postoperative day, the patient's IOP remained unchanged at 6 mmHg, but her visual acuity decreased to hand motion at 30 cm. Fundus examination revealed an RPE tear along the superior arcade vessel, extending from the optic nerve disc to the superior-temporal peripheral area, as well as bullous retinal detachment extending from the posterior pole to the inferior peripheral retina (Figures 2(a) and 2(b)). FAG demonstrated hyperfluorescence corresponding to the RPE tears (Figures 2(c) and 2(d)). No obvious retinal breaks were observed. Considering the possibility that the patient had developed RPE tears due to low IOP after filtration surgery, transconjunctival suturing of the scleral flap was performed with 10-0 nylon. Although the IOP remained low at 7 mmHg, the inferior subretinal fluids gradually decreased after the procedure, and we decided to follow up the patient. However, two months after the surgery, the RPE tears enlarged, and the retinal detachment gradually worsened. To address this, we performed not only additional transconjunctival suturing of the scleral flap but also an intracameral injection of high molecular weight ocular viscoelastic devices. Four months after the surgery, the right IOP stabilized at 12-15 mmHg, and the retina began to reattach gradually. At 12 months postoperatively, the retina in the central fovea was attached, and BCVA of the right eye improved to 0.3. However, the RPE tear persisted, and tractional retinal folds caused by subretinal proliferative tissue were observed (Figures 3(a)–3(c)).

## 3. Discussion

In general, most RPE tears are associated with vascularized PED due to age-related macular degeneration (AMD). The mechanism of RPE tears is thought to be mechanical stretching and breakage of the RPE due to the accumulation of subretinal fluids in the subretinal pigment epithelial space and increased hydrostatic pressure within the PED. While RPE tears can occur spontaneously, anti-VEGF drug therapy or photodynamic therapy can also cause RPE tears due to traction in the RPE layer associated with the contraction of choroidal neovascularization [2, 9]. However, RPE tears are a rare complication after glaucoma filtration surgery, with only a few cases reported to date. At present, neither the pathogenesis nor the treatment for this complication has been established [5, 10].

Harada et al. reported a case of RPE tears caused by uveitis after trabeculectomy (TLE) in a patient with secondary glaucoma in his 60s. Two months after TLE, SRD and RPE tears with choroidal detachment occurred at an IOP of 5 mmHg, and steroid pulse therapy was ineffective. Vitrectomy resulted in increased IOP, which temporarily improved SRD. However, SRD recurred as IOP decreased. As they considered increasing IOP to be important for treatment, transconjunctival suturing of the scleral flap was performed to increase IOP. After the procedure, the IOP increased to 12 mmHg and SRD improved without recurrence [5]. Yoshida et al. also reported a case of RPE tears 4 weeks after trabeculectomy combined with vitrectomy in a 60-year-old patient with neovascular glaucoma. Because the patient had an episode of continuous coughing caused by asthma before the onset, the authors speculated that the Valsalva maneuver led to choroidal detachment. Submacular hemorrhage was also observed; therefore, they performed vitrectomy with a tissue-type plasminogen activator. The IOP was 20 mmHg on the day after the surgery, and the choroidal detachment and SRD resolved without recurrence [11]. In a report by Cutolo et al., Ex-PRESS filtration surgery was performed on a woman in her 60s suffering from POAG. After the surgery, SRD and RPE tears with choroidal detachment developed at an IOP of 5 mmHg. One day after transconjunctival suturing of the scleral flap was performed, the choroidal detachment and SRD resolved [12]. These cases shared a common feature of RPE tears occurring alongside choroidal detachment in a low IOP environment that improved with increasing IOP. This was also observed in the present case. SRD and RPE tears with choroidal detachment developed when the postoperative IOP decreased to 6 mmHg after Ex-PRESS filtration surgery was performed on a patient in his 60s, and choroidal detachment and SRD improved when the IOP increased. Previous reports have proposed the following hypothesis regarding RPE tears and SRD after filtering surgery, which stated that choroidal circulatory failure due to low IOP causes exudate to accumulate in the suprachoroidal space [13, 14]; as a result, the retina and choroid are raised on the vitreous, and the RPE is mechanically stretched and ruptured [2]. Barrier dysfunction of the RPE causes fluid to move from the suprachoroidal space to the subretinal space, resulting in SRD [1, 5]. Based on this hypothesis, they have suggested that choroidal detachment associated with low IOP is the main triggering factor in the development of RPE tears and that it is essential to improve choroidal detachment by increasing IOP for treatment. However, although many cases of choroidal detachment after filtration surgery have been observed, RPE tears are extremely rare. The risk factors for the development of RPE tears are yet to be known.

The unique feature of this case was that the patient had multiple arteriosclerotic systemic diseases and an episode of suspected nonarteritic ischemic optic neuropathy (NAION) during follow-up for glaucoma. NAION is presumed to be caused by circulatory failure of the short posterior ciliary artery (SPCA), and arteriosclerotic diseases such as hypertension and diabetes are known to be risk factors [15]. Additionally, it has been pointed out that the SPCA, a feeding vessel of the optic disc, is also involved in blood flow in the choroid near the optic disc, and there have been many recent reports suggesting a relationship between NAION and the vascular structure of the choroid [15]. Hayreh et al. reported that the PCA supplies blood mainly to the optic disc but also to the local choroid and that barrier function of the RPE is impaired due to ischemia of the choroid [16]. In the present case, the patient had multiple atherosclerotic systemic diseases such as diabetes, hypertension, and hypercholesterolemia, and the episodes of ischemic optic neuropathy may have resulted from circulatory failure of the PCA. Circulatory failure of the PCA may have caused not only ischemia of the optic disc but also barrier dysfunction of the RPE due to ischemia of the choroid. In fact, FAG suggested a choroidal blood flow disorder on the nasal side of the optic disc, and vascular lesions in the SPCA were suspected. As a result of choroidal blood flow disorder causing dysfunction of the RPE, disruption of cell adhesion factors within the RPE may render it more susceptible to mechanical stretching. Moreover, since scattered drusen were observed in the peripheral retina, chronic inflammation of the RPE may have also influenced this condition. In patients with a background of vulnerable RPE, we speculated that choroidal detachment due to low IOP after filtration surgery exacerbated choroidal circulatory failure and barrier dysfunction of the RPE, resulting in RPE tears. With this speculation, since the RPE tears that developed in this case began on the nasal side of the optic nerve disc and progressed obliquely superior-temporal side, it may be that the primary tear occurred on the nasal side where the RPE was weakened by reduced choroidal blood flow and the tear expanded superiorly as the RPE layer was stretched by the choroidal detachment. This case indicates that if a patient has a history of ocular diseases such as choroidal blood flow disorders or drusen that lead to vulnerability to the RPE, the postoperative fall in IOP should be managed to avoid the development of choroidal detachment when performing filtration surgery. However, the relationship between RPE tears and choroidal circulatory disturbance in the background of ischemic disease is still a matter of speculation and will need to be verified using indocyanine green angiography, laser speckle fluorography, OCT angiography, and other tests in future.

Finally, in our case, it took a relatively long time for IOP to increase and stabilize after the onset of RPE tears. Therefore, it can be inferred that the subretinal fluid was absorbed slowly, resulting in the accumulation of subretinal proliferative tissue, possibly due to the scattering of RPE cells and residual tractional retinal detachment caused by the proliferative tissue. Our observations suggest that the time from the onset of RPE rupture to IOP elevation may be related to the recovery of retinal detachment and improvement of visual acuity.

## 4. Conclusion

We encountered a case of RPE tears and serous retinal detachment after Ex-PRESS filtration surgery. The patient had a history of ischemic optic neuropathy that was suspected to be related to the development of the disease. Although all of the cases reported to date have been associated with low IOP and choroidal detachment, it remains unclear which preoperative factors are associated with a high risk for the development of RPE tears. This case suggests that RPE structural vulnerability may be associated with RPE tear development due to an underlying history of diseases causing RPE dysfunction, such as choroidal perfusion disorders and drusen. However, further case studies are required to validate this.

## Data Availability

The datasets used and/or analyzed in the course of the current study are available from the corresponding author on reasonable request.

## Abbreviations

RPE: Retinal pigment epithelial
SRD: Serous retinal detachment
POAG: Primary open-angle glaucoma
IOP: Intraocular pressure
VEGF: Vascular endothelial growth factor
CSC: Central serous chorioretinopathy
BCVA: Best-corrected visual acuity
OCT: Optical coherence tomography
PED: Pigment epithelial detachment
cpRNFL: Circumpapillary retinal nerve fiber layer
FAG: Fluorescein angiography
AMD: Age-related macular degeneration
TLE: Trabeculectomy
NAION: Nonarteritic ischemic optic neuropathy
SPCA: Short posterior ciliary artery.
